# High-performance thermally-evaporated light-emitting diodes via one-step vapor purification

**DOI:** 10.1038/s41377-026-02226-4

**Published:** 2026-04-20

**Authors:** Xiang Zhang, Yuanwu Wu, Jianfeng Ou, Zixi Shen, Nian Liu, Thamraa Alshahrani, Abd. Rashid bin Mohd Yusoff, Jiang Tang, Jiajun Luo

**Affiliations:** 1 Wuhan National Laboratory for Optoelectronics (WNLO) and School of Optical and Electronic Information, Huazhong University of Science and Technology (HUST), Wuhan, Hubei China; 2 Jiufengshan Laboratory, Wuhan, Hubei China; 3 School of Microelectronics, Hubei Provincial Engineering Research Center for Wide-Bandgap Semiconductor Materials and Devices, Wuhan Textile University, Wuhan, Hubei China; 4 Department of Physics, College of Science, Princess Nourah Bint Abdulrahman University, Riyadh, Saudi Arabia; 5 Physics Department, Faculty of Science, Universiti Teknologi Malaysia, Johor Bahru, Malaysia

**Keywords:** Lasers, LEDs and light sources, Displays

## Abstract

Purity has been fundamental to the fabrication of high-performance semiconductors, spanning from precursors to reaction atmospheres. Emerging thermally evaporated perovskites involving precise control impose even stricter requirements on the purity of the vapor atmosphere. Compared to the purified solvent for the reaction atmosphere in the solution-based method, efficient and effective vapor purification remains unexplored. Here, we report one-step vapor purification for a pure atmosphere with less than one percent detrimental impurity. The in-situ residual gas analysis was employed to emphasize the complexity of the gas-phase composition and reveal the impurity reaction competition during the purification. The pre-control of the vapor atmosphere enabled a record external quantum efficiency over 20% for a thermally-evaporated perovskite light-emitting diode (PeLED) based on defect-suppressed FA_*x*_Cs_1-*x*_PbBr_3_ films. Moreover, the PeLED display panels exhibit over 5-fold and 2-fold magnification in operational and storage stability. The stability extension in OLEDs by this strategy demonstrates its universal effectiveness and commercialization potential for other types of thermally evaporated optoelectronic devices.

## Abstract (web summary)

Stable and pure atmosphere enabled by a one-step vapor purification strategy for high-performance thermally-evaporated light-emitting diodes.

## Graphical abstract

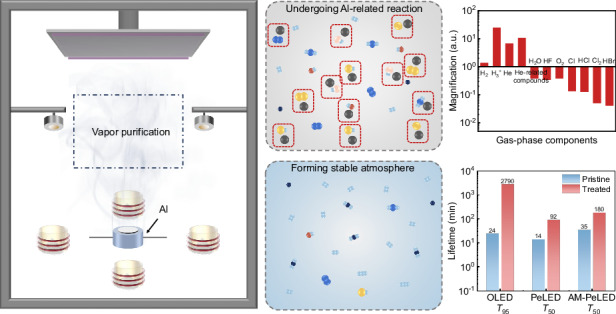

## Introduction

Low impurity doping is a critical factor for achieving high-performance devices in the semiconductor industry^1–4^. In the traditional semiconductor field, the purified materials are typically used for atmosphere control and sample preparation, providing the foundation for subsequent controllable doping^5,6^. Taking the solution-based synthesis of semiconductors as an example, low-purity solvents and precursors often lead to significant degradation of optoelectronic performance due to reactions between impurities and precursors^2,7–9^. In thermal evaporation, the vapor atmosphere is not directly determined by the purified materials, leading to a challenge for desirable devices.

Thermal evaporation has been reinvigorated for in-situ assembling optoelectronic devices, owing to the ever-increasing demand for integration^10–13^. These merits are further amplified in organic light-emitting diodes (OLEDs), contributing to their successful commercialization^14–16^. Emerging perovskite LEDs (PeLEDs) have attracted significant academic and industrial interest due to their high efficiency, pure light emission, and scalable fabrication^6,17–21^. In recent years, thermal evaporation has gained attention as a promising technique for perovskite nanocrystal films^22–26^. In 2022, Li et al. demonstrated industry-compatible, all-thermally evaporated PeLEDs, achieving a breakthrough external quantum efficiency (EQE) exceeding 16% and an active-matrix display panel^25^. These advances suggest that thermal evaporation will continue to drive next-generation LEDs, especially for high-integration advanced displays.

In OLED fabrication, the entire deposition process within a vacuum chamber only involves physical processes, due to pre-synthesized organic materials^27–29^. The vacuum level in chambers is commonly controlled, ensuring the formation of uniform films. In contrast, the thermally-evaporated perovskite films are much more complicated, including: (1) physical evaporation of precursors in the vacuum chamber; (2) chemical reactions, nucleation, and crystal growth near the substrate^25,30,31^. Clearly, the precision required for such chemical vapor deposition far exceeds that of traditional physical vapor deposition, with each step critically influencing the films’ quality^22,30,32^. This deeper understanding of the vapor deposition mechanism enlightens a reconsideration of more accurate vapor purification for a stable atmosphere^33–35^. Traditional vapor purification strategies typically rely on the vacuum pumping process, which is carried out using cascaded vacuum pumps. Despite its procedural simplicity, these methods exhibit significant limitations in both impurity removal and the attainment of low pressure. Moreover, the additional extension of vacuum pumping duration significantly increases the complexity of the equipment and the time required for vapor purification. Efficient and effective vapor purification is crucial for suppressing defects induced by impurity doping and will facilitate the development of high-performance thermally evaporated LEDs, solar cells, and other types of devices.

Herein, we demonstrate a pure vapor atmosphere with less than one percent detrimental impurity enabled by one-step vapor purification. The in-situ residual gas analysis (RGA) was employed to reveal the composition and reaction competition of the gas-phase impurity during the purification process. The impurity-suppressed atmosphere facilitated the growth of low-defect-density perovskite films, yielding thermally evaporated PeLEDs with exceptional EQE exceeding 20%. Importantly, the integrated PeLED display panels fabricated in this cleaner atmosphere demonstrate improved operational (over 5-fold) and storage (over 2-fold) stability. Additionally, a more than 100-fold enhanced operational lifetime and purer emission in thermally-evaporated blue OLEDs show the great potential of this strategy in a broader field.

## Main

### Mechanism of one-step vapor purification

**Fig. 1:**
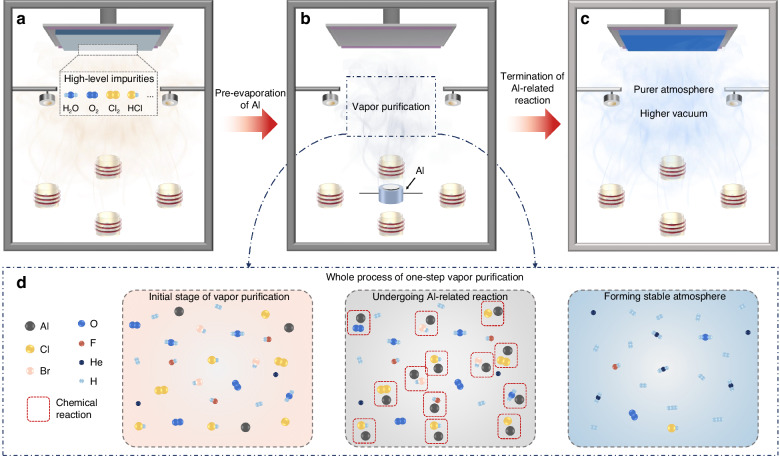
One-step vapor purification. Schematic diagram of vacuum chambers in different states, including an untreated vacuum chamber (**a**), the vacuum chamber during one-step vapor purification (**b**), and the treated vacuum chamber (**c**). **d** Whole process of one-step vapor purification with three different stages

The maintenance of a clean chamber during vapor deposition is critically important, as gase-phase impurities (e.g., H_2_O, O_2_) can lead to undesirable films and accelerated degradation of optoelectronic devices. Traditional vapor deposition, commonly used in OLEDs, mandates a high vacuum at a level of 10^−4^ Pa to ensure sufficient purity for manufacturing. Recent progress on perovskite nanocrystal films via chemical vapor deposition has fundamentally challenged the established requirements, prompting a deeper reconsideration of the chamber atmosphere. Here, in-situ RGA was performed to investigate the gas-phase components in the vacuum chamber at ~2.4 × 10^−4^ Pa^36,37^. The results reveal that the detrimental gas-phase components continue to pose non-negligible threats to device performance despite the high vacuum (Fig. 1a and Supplementary Fig. 1).

In addressing this challenge, we propose a one-step vapor purification strategy to reduce the gas-phase impurities in the vacuum chamber. This strategy achieves vapor purification of the vacuum chamber through in-situ reactions of pre-evaporated active metals, eliminating the additional operations and equipment. As an easily available metal with high chemical reactivity, aluminum (Al) is a promising candidate for the reactions with detrimental gas-phase impurities in vacuum chambers, such as Cl_2_, H_2_O, O_2,_ and others (Fig. 1b, d, and Supplementary Note 1). The reaction products primarily consist of non-detrimental gases (e.g., H_2_) and inert solid compounds (e.g., Al_2_O_3_). The gas-phase products exert negligible influence on subsequent deposition processes, while the inert solid deposits form a conformal layer on chamber walls (Fig. 1c). The chemical stabilization of the deposition environment can benefit from the pre-treatment with suppressed gas-phase impurities and side-wall re-evaporation.

**Fig. 2:**
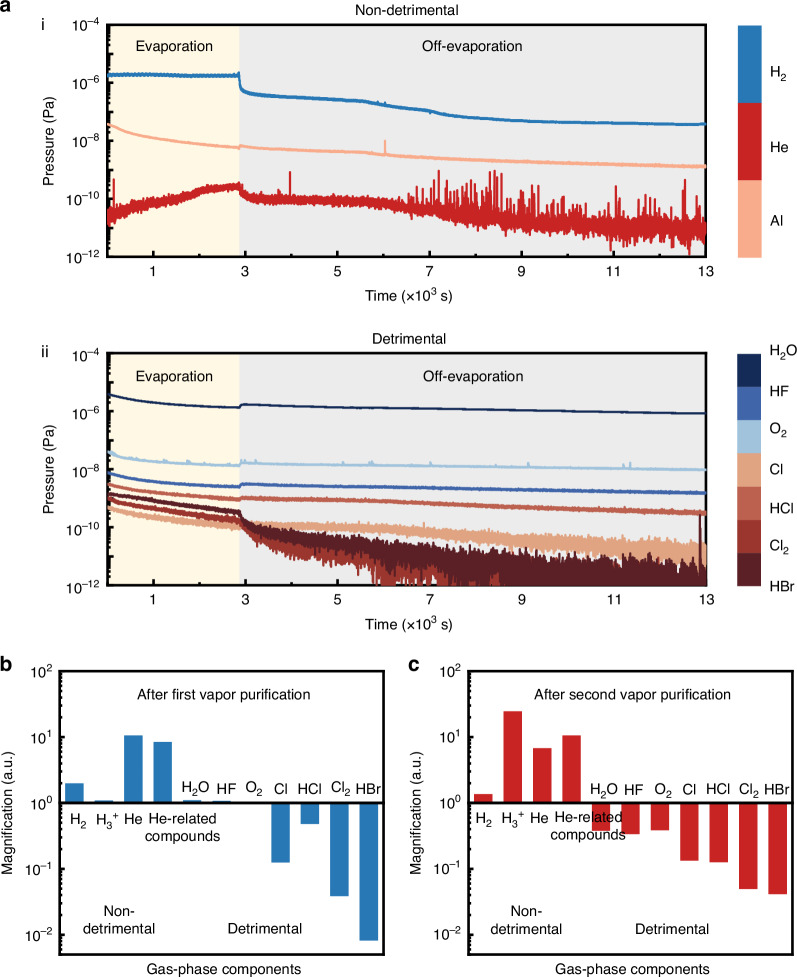
In-situ residual gas analysis. **a** Pressure variation of non-detrimental gas-phase components (**i**) and detrimental gas-phase components (**ii**) in the vacuum chamber during evaporation or off-evaporation. Magnification of the partial pressure of all gas-phase components after the first vaporpurification (**b**) and the second vapor purification (**c**)

To further investigate the mechanism of the one-step vapor purification strategy, we conducted in situ RGA measurement to monitor the dynamic reaction process (Fig. 2ai, ii). During the pre-evaporation of reactive metal (Al used here), the pressures of non-detrimental gases are maintained at relatively high levels, while the pressures of detrimental gas-phase components show a gradual decreasing trend. The primary reason is that Al-related reactions consume gaseous impurities in the vacuum chamber and generate non-detrimental gas-phase components. When the evaporation ends, as a relatively reactive gas, H_2_ will tend to form H_3_^+^ and He-related compounds, leading to a decrease in the pressures of H_2_ and He.

Additionally, Al-related reactions will continue, which can be further observed in the subsequent declines in the pressure of Al. Al reacts preferentially with Cl_2_ and HBr, leading to a rapid decrease in the partial pressures of these two gaseous impurities. When the temperature was gradually cooled down, the residual HCl and Cl^−^ will then react and combine with Al to form AlCl_3_ due to its low melting point and boiling point. However, the abnormal increase in the pressure of some detrimental gas-phase components indicates their reduced participation in ongoing reactions after off-evaporation. The RGA results after the first pre-evaporation of Al showed the nearly unchanged partial pressure of H_2_O, HF, and O_2_, while other detrimental gas-phase components are obviously reduced (the magnification of partial pressure < 1), showing the competitive reaction between Al and different detrimental gas-phase impurities(Fig. 2b and Supplementary Table 2). The sequence of competitive reaction with Al is consistent with the bond energy of gas-phase impurity molecules, while the O=O bond in O_2_ and the two H–O bonds in H_2_O delay the reaction (Supplementary Note 1 and Supplementary Table 1). Therefore, the first pre-evaporation of Al mainly removes the weakly-bonded halogen-related gas-phase impurities.

To further suppress the detrimental gas-phase impurities like H_2_O and O_2_ in the vacuum chamber, a second evaporation of Al was performed. Compared with the chamber environment after the first pre-evaporation, the partial pressure of H_2_O, HF, O_2_, and HCl (group A) was further reduced, whereas that of Cl and Cl_2_ (group B) remained almost constant (Fig. 2c and Supplementary Table 2). This primarily arises due to the continued reaction between Al and group A after group B was sufficiently consumed, further confirming the presence of competitive reactions during the pre-evaporation process. Here, the abnormal increase in the partial pressure of HBr can be attributed to the rapid decomposition of AlBr_3_ during the second pre-evaporation.

The final RGA results show that the pressures of all detrimental gas-phase components significantly decreased to no more than 10^−6^ Pa level (Fig. 2d). The pressure of detrimental gas-phase impurities is summarized to less than 1% of the total pressure (~4 × 10^−5^ Pa) within the vacuum chamber. Under this pressure, inorganic precursors (PbX_2_ and CsX) travel ballistically toward the substrate with few chances to react with residual Al. This one-step vapor purification strategy demonstrates the effective reduction of gas-phase impurities and optimized vacuum degree, enabling cleaner chambers for the subsequent fabrication of high-performance optoelectronic devices. For convenience, the pristine and treated devices correspond to the devices fabricated before or after Al pre-evaporation in discussions.

### The performance of PeLEDs

Based on the vacuum chamber with remarkable improvement of atmospheric purity, the pristine and treated perovskite films and their characterizations were conducted to understand the influence of gas-phase impurities during the vapor deposition. The photoluminescence (PL) measurements were first performed. As shown in Supplementary Fig. 2a, the unaffected perovskite composition can be proved from nearly the same emission peaks of these perovskite films at ~527 nm in PL spectra. Notably, the PL intensity is obviously enhanced in the treated film, which may indicate fewer defects caused by the undesirable atmosphere in a vacuum chamber. The average and maximum photoluminescence quantum yield (PLQY) also demonstrate the same comparison between the pristine and treated films (Supplementary Fig. 2b).

**Fig. 3:**
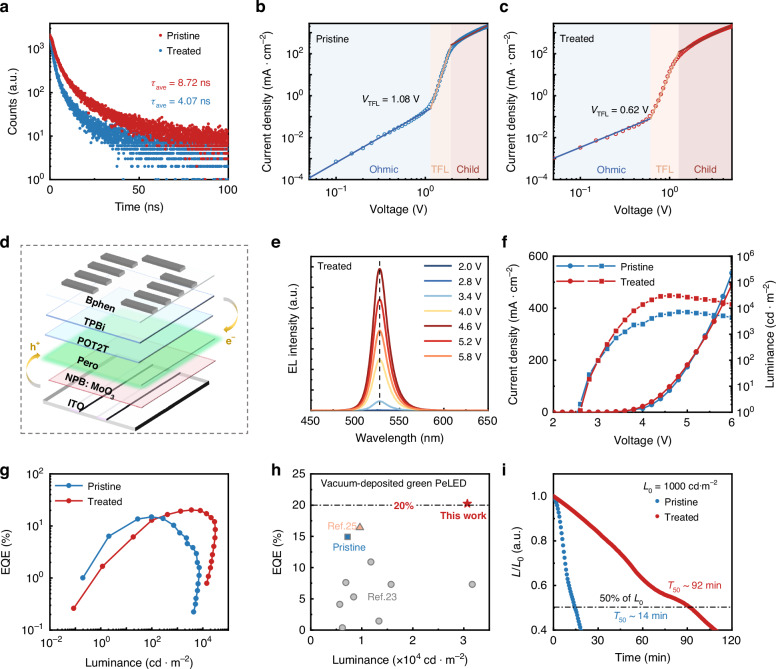
Device performance of PeLEDs. **a** TRPL spectrum of the pristine and treated films. The quality index *R*^*2*^ > 0.99 for both fittings. SCLC results of the pristine (**b**) and treated (**c**) perovskite-based devices. **d** Device structure of the pristine and treated PeLEDs. **e** The EL spectra of the treated PeLEDs under different voltages. **f** Current density (*J*) and luminance (*L*) versus voltage (*V*) curves of the PeLEDs. **g** EQE versus luminance curves of the PeLEDs. **h** Summary of EQE values reported for thermally-evaporated green PeLEDs. **i** Operational stability tests for the PeLEDs

Time-resolved photoluminescence (TRPL) measurements were carried out to investigate the carrier recombination in Fig. 3a. The TRPL spectra were fitted according to a biexponential decay equation $I(t)={I}_{0}+{A}_{1}\exp (-t/{\tau }_{1})+{A}_{2}\exp (-t/{\tau }_{2})$^24^. Average PL lifetimes (*τ*_ave_) were calculated using the equation ${\tau }_{ave}=({A}_{1}{\tau }_{1}^{2}+{A}_{2}{\tau }_{2}^{2})/({A}_{1}{\tau }_{1}+{A}_{2}{\tau }_{2})$. The *τ*_ave_ was improved from 4.07 ns (for the pristine film) to 8.72 ns (for the treated film), thus preliminarily reflecting the reduced non-radiative recombination caused by defects. Then, space charge limited current (SCLC) measurement was further conducted based on the hole-only device structure to verify the reduction in defects. The trap-filling limited voltage (*V*_TFL_) significantly decreased from ~1.08 V (the pristine device) to 0.62 V (the treated device), indicating a reduced trap density (Fig. 3b, c). Subsequently, X-ray diffraction (XRD) and scanning electron microscope (SEM) measurements were conducted to evaluate the improvements in crystal quality and surface morphology. The XRD patterns reveal enhanced diffraction peak intensities in the treated film, indicating superior crystallinity (Supplementary Fig. 3). SEM images further confirm that the treated sample exhibits a more uniform and continuous surface morphology compared to the pristine film, which is conducive to enhanced device performance (Supplementary Fig. 4).

Motivated by the improvement of perovskite films, PeLEDs were therefore fabricated. Here, the four-source co-evaporation was employed by individually loading FABr, CsBr, PbBr_2_, and TPPO. The PeLEDs are fabricated based on a lab-scale device structure for bottom emission with patterned ITO/NPB: MoO_3_/perovskite/POT2T/TPBi/Bphen/LiF/Al (Fig. 3d).

After vapor purification, the treated PeLED exhibits great EL stability with the emissive peak at 527 nm as the drive voltage increased (Fig. 3e). We then statistically analyzed the color coordinates under different voltages. The results show the nearly unchanged color coordinates, indicating the stable emission peak and FWHM (Supplementary Fig. 5a). Among them, the EL intensity under the voltage of 2.8 V is obviously lower than the others, resulting in the differences in the color coordinates. In addition, the treated PeLED achieved Commission Internationale de l'Éclairage (CIE) coordinates of (0.173, 0.775), which are close to the corner of the CIE 1931 color space with excellent monochromaticity. Meanwhile, this result significantly exceeds the green coordinates (0.21, 0.71) in the National Television Standards Committee standard and gets close to (0.170, 0.797) in the Rec. 2020 standard, showing the potential of expanded color gamut for displays with high color reproduction. Differently, the pristine PeLED exhibits a noticeable spectral shift as the voltage increases, which means that the emissive layer underwent significant degradation during operation (Supplementary Fig. 5b, c).

The current density-voltage-luminance (*J*-*V*-*L*) and *EQE*-*L* curves of the pristine and treated PeLEDs were also recorded. The treated PeLED shows a significant improvement in brightness compared to that of the pristine PeLED (Fig. 3f). Meanwhile, the current density of the treated PeLED at its maximum brightness is ~97 mA∙cm^−2^, which is significantly lower than that of the pristine PeLED (~124 mA∙cm^−2^). In addition, the treated PeLED exhibited an astonishing EQE exceeding 20% (Fig. 3g, h, and Supplementary Fig. 6), which is much higher than that of the pristine PeLED (EQE < 15%). These results indicate that the treated PeLEDs possess more efficient utilization of electrons and holes. Considering the identical device structures, the differences in the emissive layers caused by gas-phase impurities within the vacuum chamber may be the prominent reason. The operational stability for unencapsulated pristine and treated PeLEDs is further investigated inside a nitrogen-filled glove box at 25 °C, as shown in Fig. 3i. The time required for luminance to reach half of the maximum value (*T*_50_) was employed here to evaluate the operational lifetime of PeLEDs due to their intrinsic instability. At an initial brightness of 1000 cd∙m^−2^, the *T*_50_ of the treated PeLED has significantly increased to over 90 minutes, which is over 5-fold higher than that of the pristine PeLED.

### The performance of the integrated PeLEDs display panel

To demonstrate the potential of this strategy in integrated production lines, active-matrix (AM) PeLED display panels were fabricated after one-step vapor purification. Due to the opaque silicon-based backplane, the commonly used bottom-emitting structure in the laboratory needs to be modified into a top-emitting structure. Here, a semi-transparent thin Ag electrode (15 nm) was employed to replace the thick electrode (100 nm Ag in OLED and 100 nm Al in PeLED). For the match between the thin electrode and the electron transport layer, a thin interlayer of Cs_2_CO_3_ (1 nm) was used. By sequentially depositing the transport layers, emissive layers, and semi-transparent electrodes onto the prepared circuit backplane, AM-PeLED display panels were fabricated on the CMOS driver (Supplementary Fig. 7).

Through the well-programmed software, each pixel of the AM-PeLED display panels can be independently controlled. We separately drove and tested the luminance of nine regions on the treated AM-PeLED display panel. The results showed that the average luminance of the nine regions was 1065 cd∙m^−2^, with less than 10% deviation between the maximum or minimum values and the average value. Here, uniformity (*U*) was defined as the equation $U=1-\frac{{L}_{\max }-{L}_{\min }}{{L}_{\max }+{L}_{\min }}$ using the maximum and minimum values of the nine regions. The calculated *U* reaches nearly 95% for the treated AM-PeLED display panel, demonstrating great potential for prototype demonstrations.

**Fig. 4:**
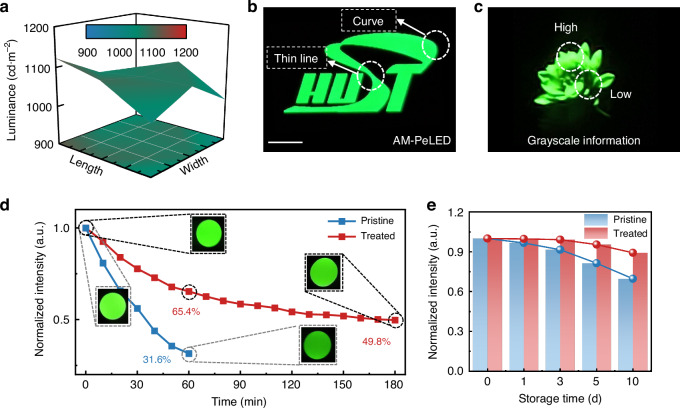
Demonstration of AM-PeLED display panels. **a** Luminance of the nine districts on an AM-PeLED display panel. **b** Static image demonstrated by AM-PeLED display panel. Scale bar, 2 mm. **c** Image of a blooming flower with grayscale information. Operational stability (**d**) and the storage stability (**e**) of AM-PeLED display panels

Then, high-definition multimedia interface (HDMI) was used to expand the display screen to demonstrate high-definition images on a display panel. Figure 4b shows a display image ‘HUST’ with intricate curves and thin lines. Figure 4c shows a screenshot of a blooming flower with grayscale information. Benefiting from the highly integrated circuit backplane, the drive currents of all pixels can be adjusted to achieve continuous and accurate grayscale.

It should be emphasized that the display panel should maintain stable operation for future applications, which remains a considerable challenge, especially for AM-PeLED display panels. Therefore, the operational lifetime of AM-PeLED is precisely measured in a darkroom environment. Due to the aperture limitation of the detector, the practical radius of the light spot was set to 2.5 mm to record the intensity decline of the image. The fabricated AM-PeLED display panels without the atmospheric control exhibit rapid declines (~68.5%) within one hour (Fig. 4d). In contrast, the intensity decline of the treated AM-PeLED display panel could be reduced to < 35% in one hour. The final *T*_*50*_ of the treated AM-PeLED display panel was measured to 180 min, which is over five-fold magnification compared to the pristine (35 min). Notably, the intensity decline of the AM-PeLED display panel stems from the degradation of the perovskite film and the inevitable thermal effect under operation. Due to the superior heat dissipation capability of the silicon-based backplane, the negative impacts on PeLEDs caused by thermal effects are mitigated for a better operational lifetime compared to the prototype PeLEDs on glass/ITO. The difference in the operational lifetimes of the pristine and treated AM-PeLED display panels can be attributed to the more stable perovskite thin films and more efficient charge injection enabled by the vapor purification, which was revealed in the previous section. The enhanced uniformity of the treated AM-PeLED display panel also indicates better image presentation in the terminal application.

In addition, the storage stability of AM-PeLED display panels was also recorded. The introduction of excessive impurities will aggravate the films’ degradation during storage, thereby deteriorating device performance, which is the main reason for the storage instability of the pristine AM-PeLED display panel. After ten days of storage, the emission of the AM-PeLED display panel was degraded to less than 70% of its initial value. For the treated, the intensity decline was reduced to ~10%, indicating the significantly suppressed degradation caused by impurities (Fig. 4e).

### The extended verification in OLEDs

Encouraged by the results in PeLEDs and AM-PeLEDs, OLEDs and their AM display panels were fabricated for further understanding. Although the requirement for atmosphere is much lower in OLED fabrication processes, the suppression of H_2_O and O_2_ by vapor purification may also contribute to the improvement of OLEDs, especially in extending lifetime. Considering the prominent stability issues of current blue organic emissive materials^9^, therefore, we firstly constructed the blue OLEDs based on a commercial device structure with commercial materials (Supplementary Fig. 8). We fabricated four pristine OLEDs and recorded the device performance under a uniform current variation step of 2.5 mA∙cm^−2^ from 2.5 mA∙cm^−2^ to 25 mA∙cm^−2^, as commercial OLED devices are controlled by current (Supplementary Fig. 9 and 10). The results show that four pristine OLEDs exhibit nearly identical device performance, indicating the good reproducibility of these devices.

**Fig. 5:**
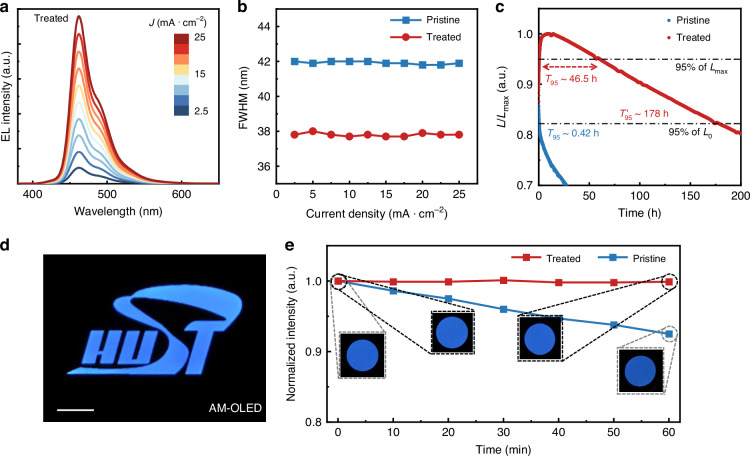
Performance of OLEDs and AM-OLEDs. **a** The EL spectra of the treated OLEDs from 2.5 mA∙cm^−2^ to 25 mA∙cm^−2^. **b** The FWHMs of the pristine and treated OLEDs from 2.5 mA∙cm^−2^ to 25 mA∙cm^−2^. **c** Lifetime of the pristine and treated OLEDs at 25 mA∙cm^−2^. **d** Static image demonstrated by an AM-OLED display panel. Scale bar, 2 mm. **e** Operational stability of AM-OLED display panels

Subsequently, we fabricated four treated OLEDs with stable blue emission (Fig. 5a and Supplementary Fig. 10). It can be found that the treated devices exhibit nearly unchanged values to those of the pristine ones in the current density-voltage (*J*-*V*), luminance-voltage (*L*-*V*) curves, and external quantum efficiency-current density (*EQE*-*J*). Although the electroluminescence (EL) spectra exhibit almost the same trend, the full width at half maximums (FWHMs) of the treated OLEDs were measured to ~38 nm, which is approximately 4 nm less than those of the pristine ones from 2.5 mA∙cm^−2^ to 25 mA∙cm^−2^ (Fig. 5b). Since the organic materials were synthesized in advance, the detrimental gas-phase components in the vacuum chamber mainly participate in the degradation process after the device preparation is completed. Here, the narrower FWHMs can be attributed to the suppressed exciton quenching after vapor purification, thus ensuring more intrinsic luminescence.

The lifetime measurement was then conducted for the pristine and treated OLEDs at 25 mA∙cm^−2^. Notably, the two OLEDs exhibit completely different trends in relative brightness changes over time (*L*/*L*_*0*_-*T*). The brightness of the pristine OLED will decrease directly after being turned on, while the brightness of the treated OLED will increase to its peak within a few hours and then start to decline. The main reason for the direct degradation of the OLEDs can be attributed to the acceleration of exciton quenching caused by the detrimental gas-phase impurities in the vacuum chamber, especially the H_2_O and O_2_. Instead, the positive effect may be dominated at the beginning of the aging process after the gas-phase impurities were removed, resulting in the initially increased brightness. Here, we define two indicators (*T*_95_ and *T*’_95_) to describe the lifetime, including the time required for luminance to reach 95% of the maximum value (*T*_95_) and the time required for luminance to reach 95% of the initial value (*T*’_95_). The *T*_95_ and *T*’_95_ of the pristine OLED are consistent due to the direct decline of brightness, reaching to ~0.4 hours (Fig. 5c). In contrast, the treated OLED exhibits a significantly extended lifetime with its *T*_95_ reaching up to ~ 46.5 h and *T*’_95_ reaching up to 178 h (Fig. 5c). The results show a more than 100-fold increase in operational lifetime compared to the pristine OLED, indicating the positive effect of dramatically reduced H_2_O and O_2_ by one-step vapor purification.

The AM-OLED display panel was also demonstrated for verification from the perspective of a prototype. The static image with similar lines displayed by AM-OLEDs can also be observed (Fig. 5d). For the operational lifetimes, the treated AM-OLED display panel exhibits obvious improvements with nearly unchanged luminance in one hour, while the pristine AM-OLED display panel shows a 7.5% intensity decline (*T*_95_ < 40 min) (Fig. 5e). These results (Supplementary Table 3 and Table 4) further demonstrate the universality and effectiveness of this vapor purification strategy in other thermally-evaporated optoelectronic devices.

## Conclusion

In summary, we focus on the atmosphere within the vacuum chamber and report a one-step vapor purification method. In-situ RGA characterization has demonstrated the fundamental process and its effectiveness in suppressing the detrimental gas-phase components, enabling them to decrease to below less than one percent of the total pressure. Benefiting from a purified vacuum chamber, significantly suppressed defect generation in vapor-deposited perovskite films slows the non-radiative recombination, enabling the EQE of green thermally-evaporated PeLEDs to exceed 20% for the first time. Moreover, the enhanced operational and storage stability of the integrated display panels indicates the commercial potential for practical application. Finally, we also demonstrate the extended employment of this strategy in OLEDs with a more than 100-fold increase in operational lifetime for blue OLEDs. This work provides in-depth insights and considerations on atmosphere and gas-phase reactions for universal high-performance optoelectronic devices.

## Method

### Materials

Formamidinium bromide (FABr, 99.999%) was purchased from Great Cell Solar Materials. Cesium bromide (CsBr, 99.999%), lead bromide (PbBr_2_, 99.999%), TPPO (98%), and Cs_2_CO_3_ (99.995%) were purchased from Sigma-Aldrich. POT2T (99.5%), TPBi (>99%), Bphen (99%), and NPB (>99%) were purchased from Xi’an Yuri Solar Co., Ltd. Lithium fluoride (LiF, 99.9%) and molybdenum trioxide (MoO_3_, 99.9%) were purchased from Aladdin. Metallic aluminum (Al, 99.99%) and silver (Ag, 99.99%) were purchased from Zhong Nuo Advanced Material Technology. All chemicals were used without further purification.

### Pre-evaporation of Al for vapor purification

Firstly, a certain degree of vacuum (<10^−3^ Pa) needs to be ensured before pre-evaporation. The sources in the vacuum chambers were separately calibrated to obtain the correct scale factors for deposition rate. Subsequently, the ceramic crucible is heated by increasing the current to achieve controllable evaporation of aluminum. When the deposition rate of aluminum becomes stable, the baffle can be opened to ensure that the fully cleaned vacuum chambers. Throughout the pre-evaporation process, the deposition rate of aluminum needs to be maintained at less than 0.3 Å∙s^−1^. A slow evaporation rate adopted here aims at minimizing Al consumption. The first pre-evaporation is completed when the pressure in the vacuum chambers is reduced by an order of magnitude compared to the initial state. The process of the second pre-evaporation maintains the same duration as the first one.

### Thermally-evaporated OLEDs and PeLEDs fabrication

The patterned ITO-coated glasses were cleaned using detergent, acetone, deionized water, and ethyl alcohol by sonication, and then treated with oxygen plasma for 15 min. The two vacuum chambers are separated for the preparation of the organic films and perovskite films. The preparation of OLED is completed by sequential evaporation of functional organic layers and emissive organic layers, among which the doped films are realized by dual-source co-evaporation. The specific OLED structure is as follows: HT: PD (10 nm, 3%)/HT (70 nm)/BH: BD (25 nm, 3%)/ET: EI (25 nm, 1:1)/Yb (1 nm)/Ag (100 nm). The preparation of PeLED is completed by sequential evaporation of functional organic layers and emissive perovskite films, among which the perovskite films are realized by four-source co-evaporation. The specific PeLED structure is as follows: NPB: MoO_3_ (45 nm, 10%)/perovskite (25 nm)/POT2T (3 nm)/TPBi (7 nm)/Bphen (30 nm)/LiF (1 nm)/Al (80 nm). Herein, the thermal evaporator (QHV-R197, Shenyang Qihui Vacuum Technology) was embedded in the glove box.

### Defect-related characterizations of perovskite film

Photoluminescence characterizations: The steady-state photoluminescence spectra were recorded using a Fluorolog-QM (HORIBA) with a monochromatized xenon lamp as the excitation source. Time-resolved photoluminescence (TRPL) decay was also recorded using a Fluorolog-QM (HORIBA) with a 374-nm-wavelength pulse laser.

Photoluminescence quantum yield characterizations: The absolute PLQYs were determined using a HiYield-PL spectrofluorometer with a calibrated integrating sphere from Oriental Spectra.

Space-charge-limited current measurements: The trap-filled limit voltage was obtained by the dark current-voltage characteristics of the hole-only devices in the device architecture of ITO/NPB: MoO_3_/perovskite/MoO_3_/LiF/Al through a computer-controlled Keithley 2400 source meter.

### Perovskite film characterizations

X-ray diffraction measurements: The XRD results were recorded with a XRD-6000 X-ray diffractometer (Shimadzu) with Cu K α radiation (λ = 1.54 Å). Scanning electron microscope measurements: The morphology of the perovskite films was examined by a GeminiSEM 300 from Carl Zeiss AG.

### Device characterizations

All OLED and PeLED device characterizations were performed in a nitrogen-filled glove box. The current density versus voltage, luminance versus voltage, EQE versus luminance, and operating lifetime curves were recorded simultaneously on a commercial measurement system (XPQY-EQE, Guangzhou Xi Pu Optoelectronics Technology) equipped with an integrating sphere and a photodetector array. This measurement system had been calibrated by halogen lamps metered by the National Institute of Standards and Technology (NIST). The emitting area of OLEDs and PeLEDs is 0.04 cm^2^, as defined by the overlapping area of ITO and top electrodes.

### Thermally-evaporated display panel fabrication

The integrated backplanes (Si (110)) were provided by Dr. Liu. The working area of the panel covers more than nine hundred thousand pixels. For the backplanes, the clean steps were simplified to using ethyl alcohol, deionized water, and ethyl alcohol by rinsing. Then, the backplanes were treated with oxygen plasma for 15 min and transferred into the vacuum chambers for the direct integration of OLEDs and PeLEDs. The bottom substrates of each pixel pit on the backplanes were filled with a total reflection electrode structure of ITO/Ag/ITO in advance. The opaque top electrodes, therefore, were replaced by semi-transparent electrodes to realize top-emission. The shadow masks were customized for accurate evaporation of OLEDs and PeLEDs on the working area of the integrated backplanes.

## Supplementary information

Supplementary Information

## Data Availability

The data that support the plots within this paper and other findings of this study are available from the corresponding author upon reasonable request.
