# Supplementary material for: High-performance thermally-evaporated light-emitting diodes via one-step vapor purification
Source: Light Sci Appl. 2026 Apr 20;15:210. doi: 10.1038/s41377-026-02226-4 (PMC13096348; doi:10.1038/s41377-026-02226-4)
Supplement: Supplementary file 1 — Supplementary Information [file 41377_2026_2226_MOESM1_ESM.docx]

**Supplementary Information**

**High-performance vapor-deposited light-emitting diodes via one-step vapor purification**

Xiang Zhang^1, 5, †^, Yuanwu Wu^1, †^, Jianfeng Ou^1^, Zixi Shen^1^, Nian Liu^2^, Thamraa Alshahrani^3^, Abd. Rashid bin Mohd Yusoff^4^, Jiang Tang^1, 5^, Jiajun Luo^1, 5,^ *

^1^ Wuhan National Laboratory for Optoelectronics (WNLO) and School of Optical and Electronic Information, Huazhong University of Science and Technology (HUST), 1037 Luoyu Road, Wuhan, Hubei 430074, P. R. China.

^2^ School of Microelectronics, Hubei Provincial Engineering Research Center for Wide-Bandgap Semiconductor Materials and Devices, Wuhan Textile University, Wuhan, Hubei 430200, China.

^3^ Department of Physics, College of Science, Princess Nourah Bint Abdulrahman University, Riyadh, 11671, Saudi Arabia.

^4^ Physics Department, Faculty of Science, Universiti Teknologi Malaysia, Johor Bahru, Malaysia

^5^ Jiufengshan Laboratory, Wuhan, Hubei 430078, China.

* The author to whom correspondence should be addressed: luojiajun@hust.edu.cn

† These authors contributed equally to this work.

**Supplementary Note 1. The reaction between gas-phase impurities and aluminum.**

Pre-evaporation of aluminum in vacuum chambers for atmospheric control involves multiple reaction processes, which are as follows:

3Cl_2_ + 2Al → 2AlCl_3_

6HCl + 2Al →2AlCl_3_ + 3H_2_
6HBr + 2Al → 2AlBr_3_ + 3H_2_
6HF + 2Al → 2AlF_3_ + 3H_2_

3O_2_ + 4Al → 2Al_2_O_3_

3H_2_O + 2Al → Al_2_O_3_ + 3H_2_


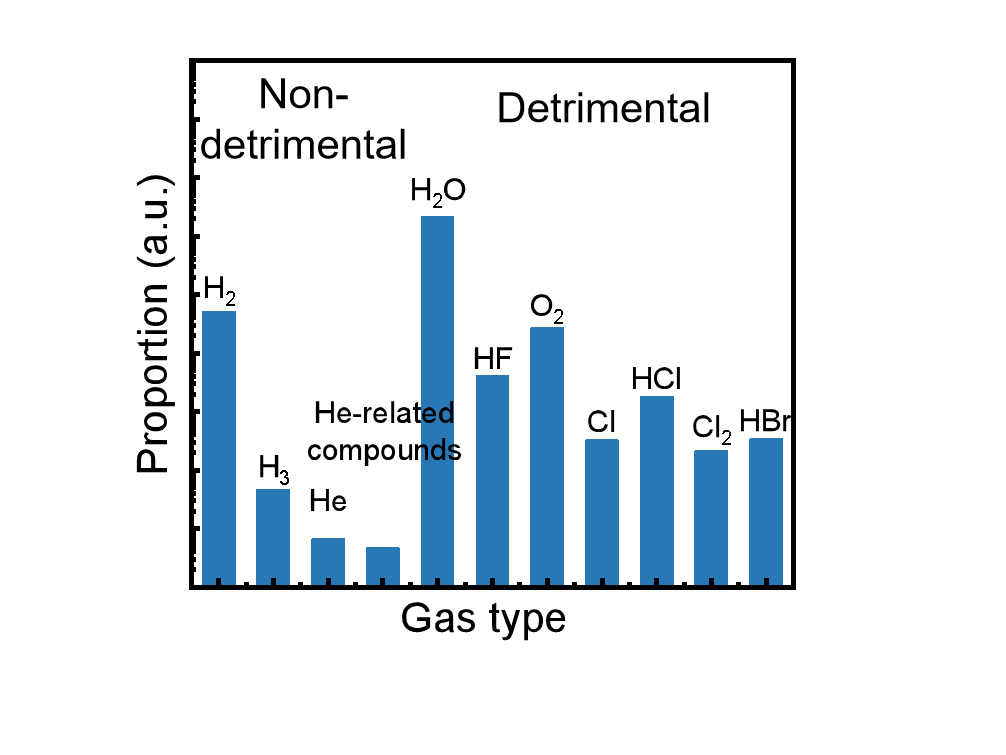


**Supplementary Fig. 1 | The partial pressure of gas-phase components in the vacuum chamber.** The detrimental gas-phase components can not be negligible, thereby posing threats to device performance despite the high vacuum.


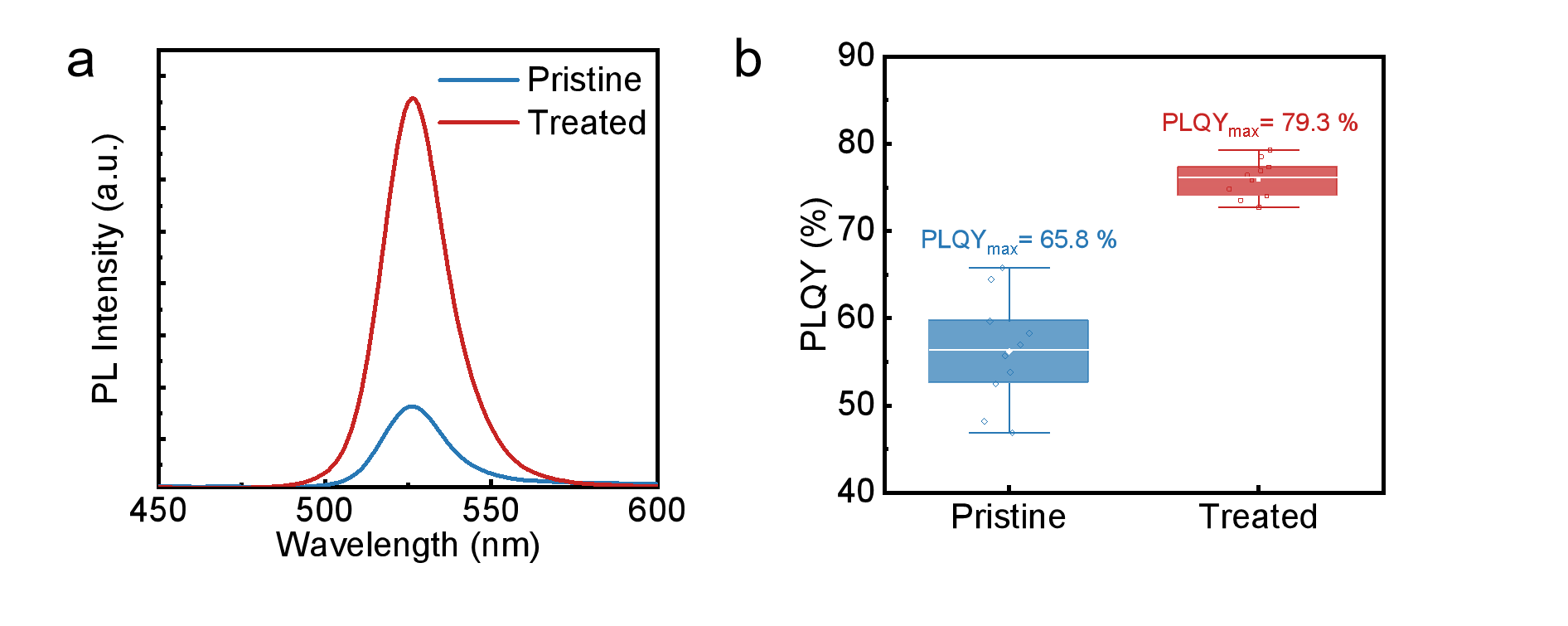


**Supplementary Fig. 2 | The PL-related characteristics of perovskite films.** **a** PL and absorbance spectra of the pristine and treated films. **b** Photoluminescence quantum yields of the pristine and treated films. The average PLQY is 56.2% and 75.9% for pristine and treated films, respectively.


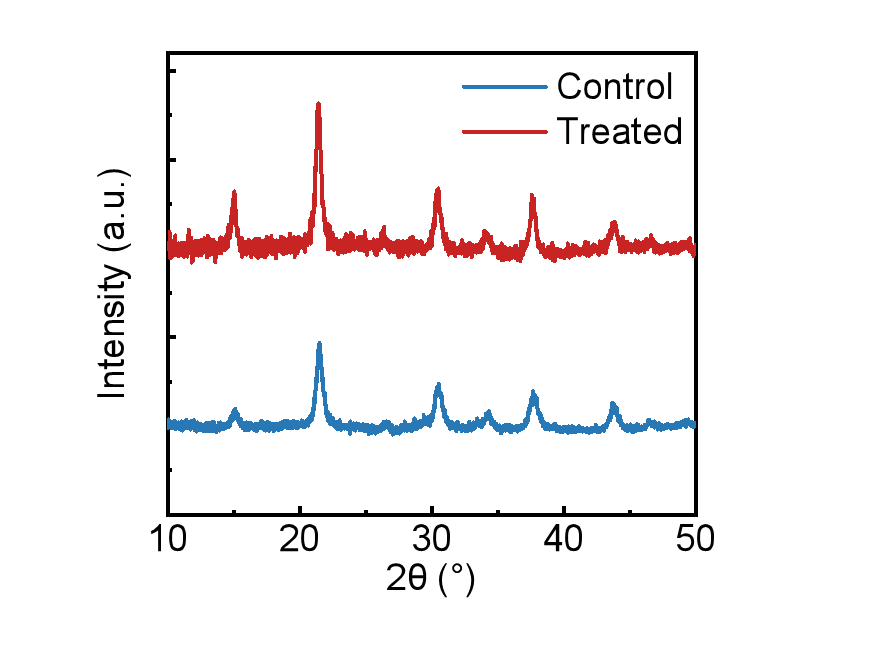


**Supplementary Fig. 3 | The XRD spectra of the pristine and treated films.** The treated perovskite films exhibit enhanced diffraction peak intensities compared to the pristine ones.


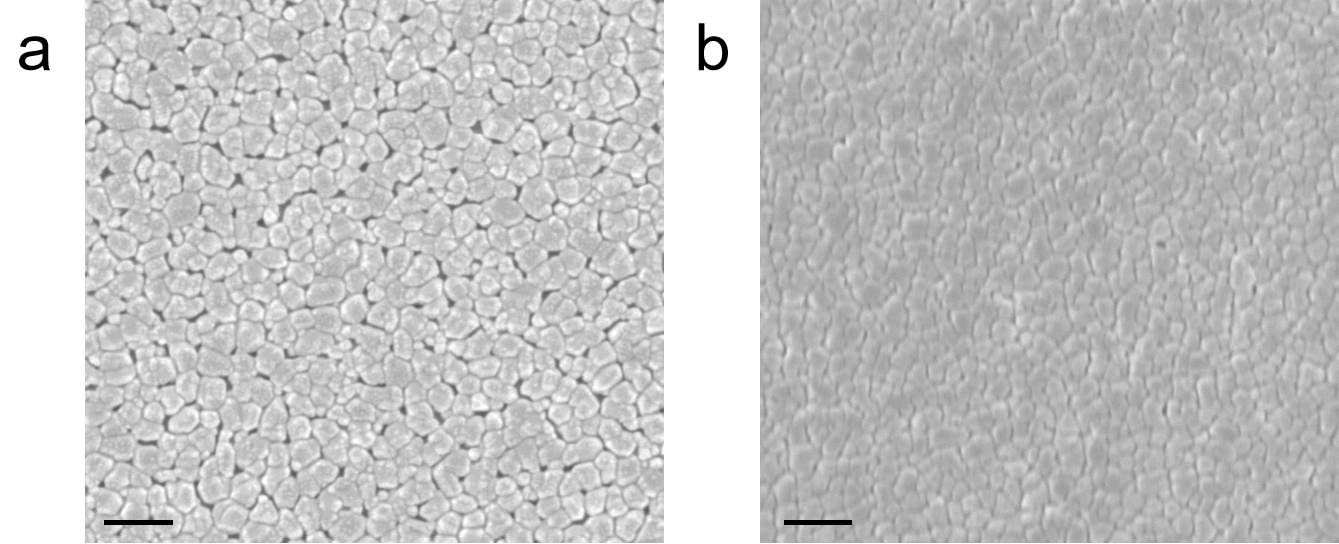


**Supplementary Fig. 4 | The XRD spectra of the pristine (a) and treated (b) films.** Scale bar, 100 nm. The crystal quality and surface morphology were improved after the vapor purification.


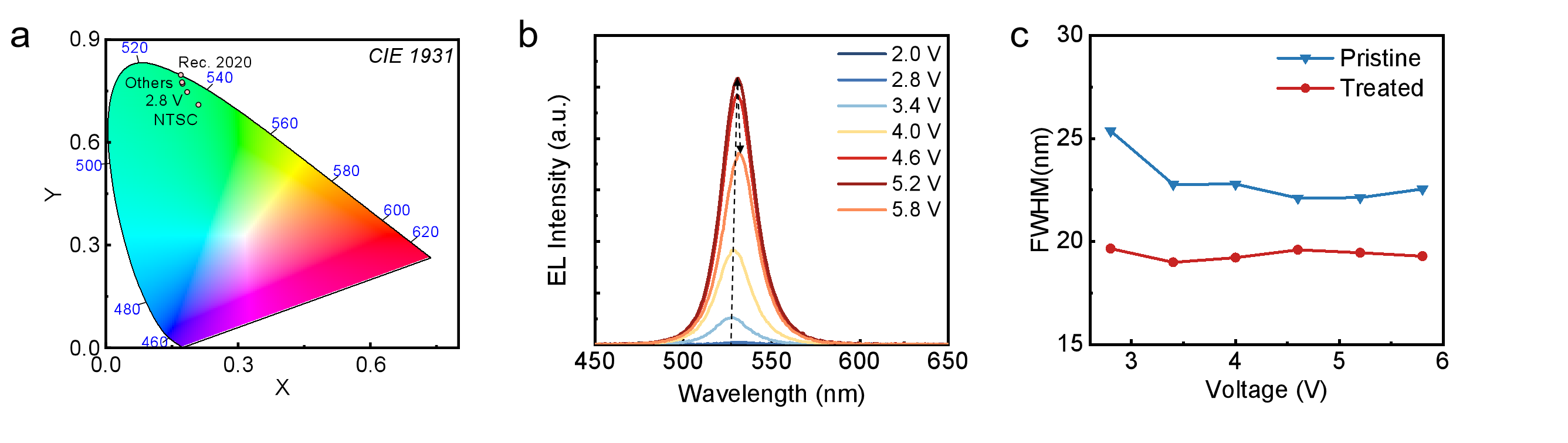


**Supplementary Fig. 5 | Electroluminescence spectra and FWHM of PeLEDs.** **a** The color coordinates of the treated PeLED under different voltages in the CIE 1931 color space. **b** The EL spectra of the pristine PeLEDs under different voltages. **c** The FWHMs of the pristine and treated PeLEDs under different voltages. The abnormal FWHM of the pristine PeLED at 2.8 V is attributed to the relatively low luminance after it was turned on. In contrast, the FWHM of the treated PeLED demonstrated more consistent results due to the significantly enhanced radiative recombination.


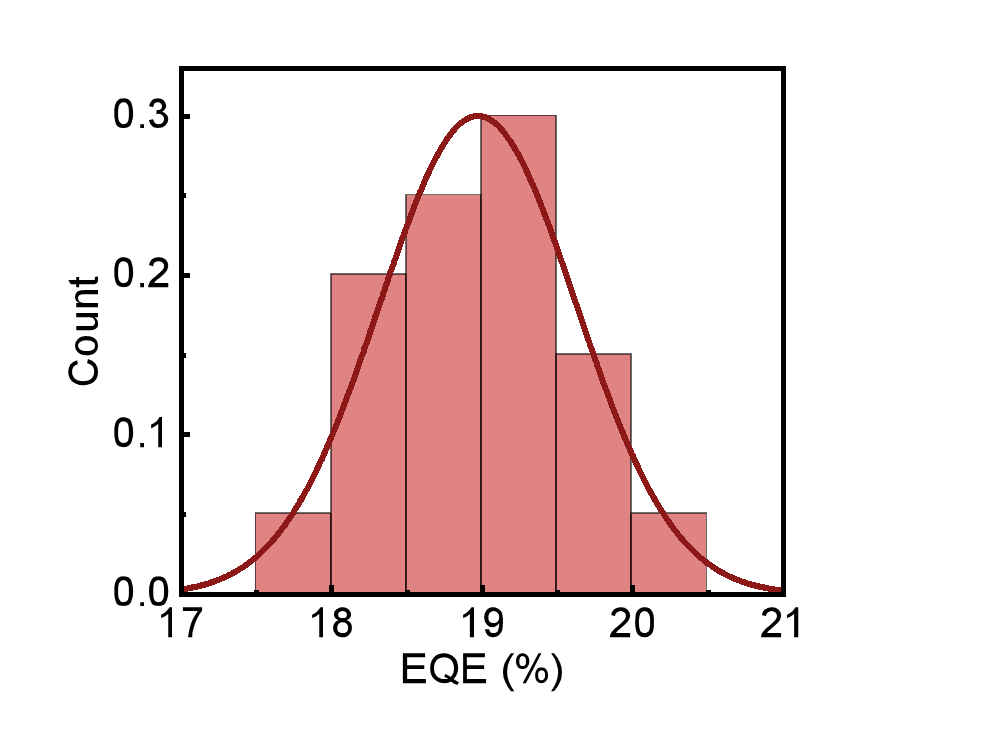


**Supplementary Fig. 6 | EQE histograms of the treated PeLEDs (20 devices).**


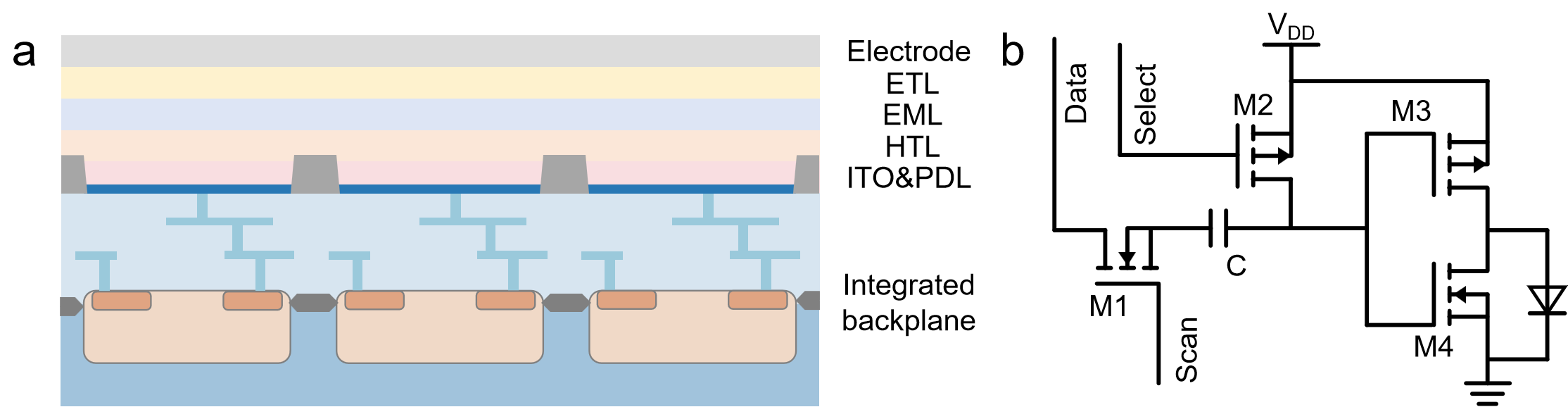


**Supplementary Fig. 7 | The pixel structure of the AM display panel. a, b** Cross-section schematic diagram of monochromatic pixels (**a**) and their basic unit of the driving circuit (**b**).


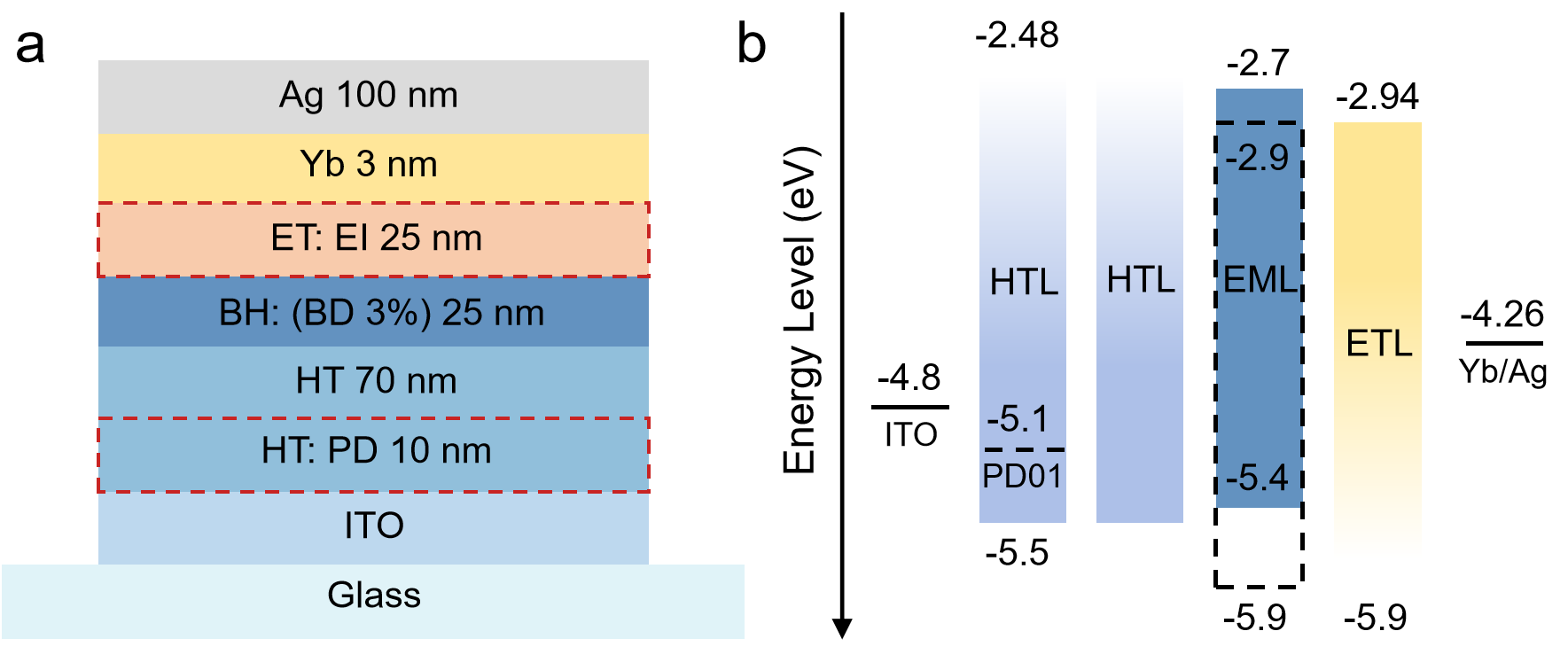


**Supplementary Fig. 8 | Detailed device structure of commercialized OLEDs.** **a, b** Schematic diagram of device architecture (**a**) and energy levels (**b**) for the commercialized OLEDs.


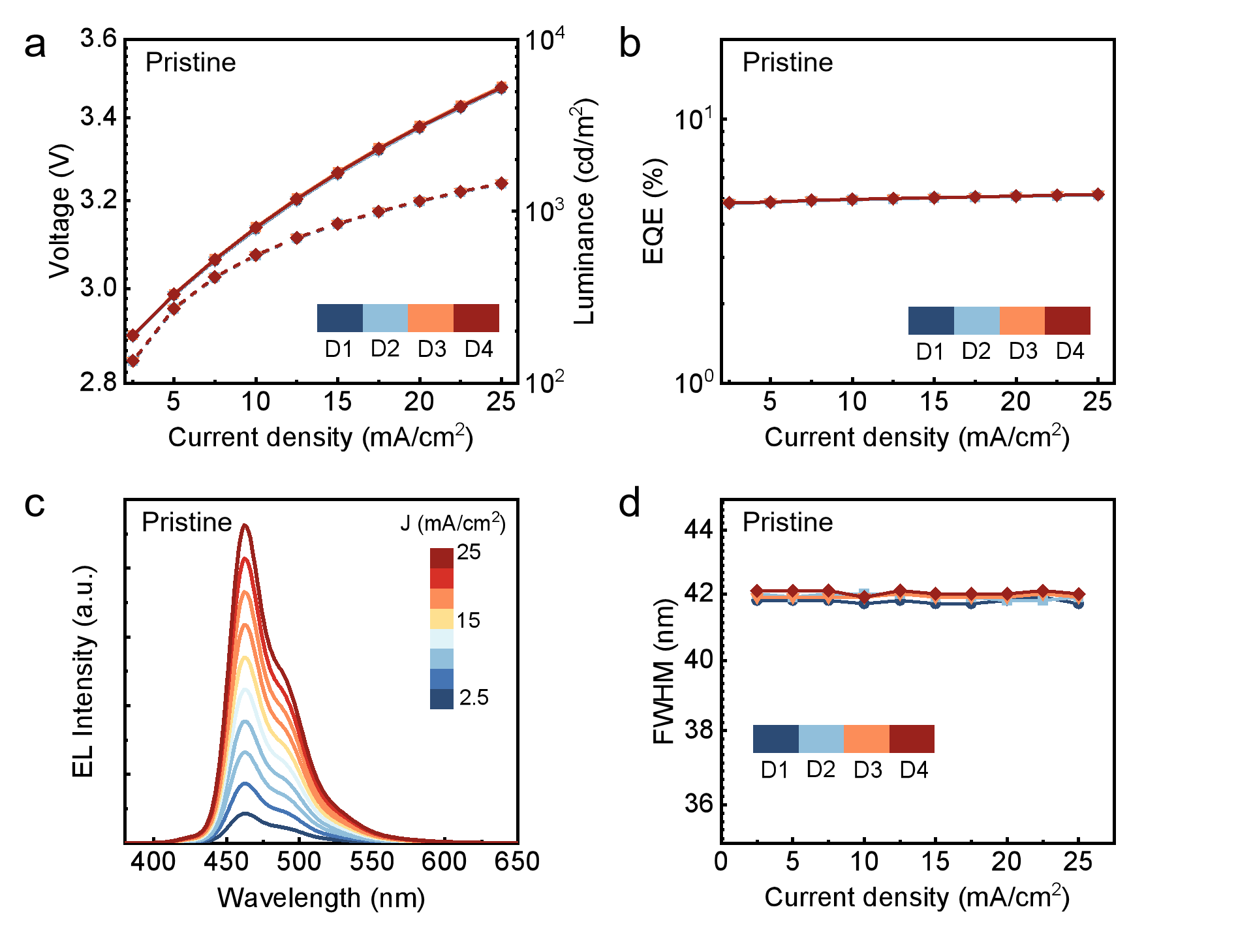


**Supplementary Fig. 9 | The detailed device performance of the pristine OLEDs from 2.5 mA∙cm^−2^ to 25 mA∙cm^−2^.** **a, b** The voltage−current density−luminance (*V*−*J*−*L*) (**a**) and EQE−current density (EQE−*J*) (**b**) of the pristine OLEDs. **c** The EL spectra of the pristine OLEDs. **d** The FWHMs of the pristine OLEDs. D1, D2, D3, and D4 represent four different pristine OLEDs.


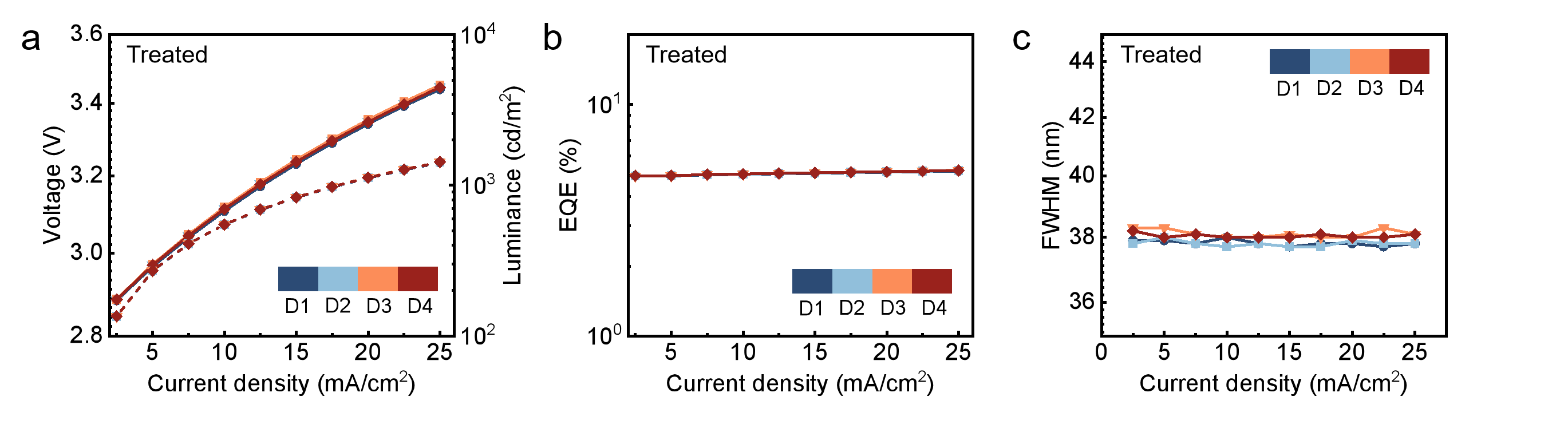


**Supplementary Fig. 10 | The detailed device performance of the treated OLEDs from 2.5 mA∙cm^−2^ to 25 mA∙cm^−2^.** **a, b** The voltage−current density−luminance (*V*−*J*−*L*) (**a**) and EQE−current density (EQE−*J*) (**b**) of the treated OLEDs. **c** The FWHMs of the treated OLEDs. The results show that the good repeatability of the OLEDs is achieved with the commercialized device structures. D1, D2, D3, and D4 represent four different treated OLEDs.

**Supplementary Table 1. The detailed bond energy of different bonds in gas-phase impurities.**

| Bond | Bond energy (kJ/mol) |
| --- | --- |
| Cl–Cl | 243 |
| H–Cl | 366 |
| H–Br | 431 |
| O=O | 498 |
| H–F | 565 |
| H–O | 464 |

**Supplementary Table 2. The detailed magnification of the partial pressure in the vacuum chamber with or without vapor purification.**

|  | The magnification of the partial pressure of gas-phase impurities | | | | | | |
| --- | --- | --- | --- | --- | --- | --- | --- |
|  | H_2_O | HF | O_2_ | Cl | HCl | Cl_2_ | HBr |
| 2/1 | 1.09 | 1.07 | 0.98 | 0.13 | 0.49 | 0.04 | 0.01 |
| 3/1 | 0.38 | 0.32 | 0.39 | 0.13 | 0.13 | 0.05 | 0.04 |

*Labels 1, 2, and 3 correspond to the original state, the state after the first Al pre-evaporation, and the state after the second Al pre-evaporation, respectively. The ratios 2/1, 3/1, and 3/2 represent comparative magnifications between these states. A magnification value below 1 indicates a reduction in impurity concentration.

**The total chamber pressure remained similar after the first and second Al pre-evaporation cycles, stabilizing at approximately 4 × 10^-5^ Pa.

**Supplementary Table 3. The detailed results of pristine and treated OLEDs and PeLEDs.**

| **Device** | | **EQE (%)** | **T_95_** | **T_50_** | **FWHM (nm)** |
| --- | --- | --- | --- | --- | --- |
| **OLED** | Pristine | ~ 5 | 0.4 h | - | ~ 42 |
|  | Treated | ~ 5 | 46.5 h | - | ~ 38 |
| **PeLED** | Pristine | 14.9 | 2.5 min | 14 min | ~ 22 |
|  | Treated | 20.2 | 10 min | 92 min | < 20 |

**Supplementary Table 4. The list of PeLED performance based on different methods.**

| **Perovskite** | **EQE** | **T_50_ lifetime** | **Demo** | **Method** | **Reference** |
| --- | --- | --- | --- | --- | --- |
| Quasi-2D | 30.84% | 288 min @ 100 cd∙m^−2^ | - | Spin coating | 1 |
| Bulk | 25.85% | 1080 min @ 100 cd∙m^−2^ | Large-area device | Blade coating | 2 |
| Quantum dots | 21.73% | 177 min @ 100 cd∙m^−2^ | Printed Logo | Ink-jet printing | 3 |
| Nanocrystal | 16.4% | 3578 min @ 100 cd∙m^−2^ | Active-matrix displays | Thermal evaporation | 4 |
| Nanocrystal | 20.23% | 92 min @ 1000 cd∙m^−2^ | Active-matrix displays | Thermal evaporation | This work |

References:

1 Dong, C. *et al.* High-Performance Green Light-Emitting-Diode Enabled by Simultaneous Phase Engineering and Crystallization Regulation. *Advanced Functional Materials* **35**, 2502662, doi:<https://doi.org/10.1002/adfm.202502662> (2025).

2 Chen, G. *et al.* Regulation of nucleation and crystallization for blade-coating large-area CsPbBr_3_ perovskite light-emitting diodes. *Science Bulletin* **70**, 212-222, doi:<https://doi.org/10.1016/j.scib.2024.10.022> (2025).

3 Lu, W. *et al.* Multifunctional Dithiol Crosslinking Additive Enables Highly Efficient and Stable Inkjet-Printed Perovskite Quantum Dot Light-Emitting Diodes. *Advanced Materials*, e15555, doi:<https://doi.org/10.1002/adma.202515555> (2025).

4 Li, J. *et al.* Efficient all-thermally evaporated perovskite light-emitting diodes for active-matrix displays. *Nature Photonics* **17**, 435-441, doi:10.1038/s41566-023-01177-1 (2023).
